# Accelerated discovery of stable lead-free hybrid organic-inorganic perovskites via machine learning

**DOI:** 10.1038/s41467-018-05761-w

**Published:** 2018-08-24

**Authors:** Shuaihua Lu, Qionghua Zhou, Yixin Ouyang, Yilv Guo, Qiang Li, Jinlan Wang

**Affiliations:** School of Physics, Southeast University, Nanjing, 211189 China

## Abstract

Rapidly discovering functional materials remains an open challenge because the traditional trial-and-error methods are usually inefficient especially when thousands of candidates are treated. Here, we develop a target-driven method to predict undiscovered hybrid organic-inorganic perovskites (HOIPs) for photovoltaics. This strategy, combining machine learning techniques and density functional theory calculations, aims to quickly screen the HOIPs based on bandgap and solve the problems of toxicity and poor environmental stability in HOIPs. Successfully, six orthorhombic lead-free HOIPs with proper bandgap for solar cells and room temperature thermal stability are screened out from 5158 unexplored HOIPs and two of them stand out with direct bandgaps in the visible region and excellent environmental stability. Essentially, a close structure-property relationship mapping the HOIPs bandgap is established. Our method can achieve high accuracy in a flash and be applicable to a broad class of functional material design.

## Abstract (web summary)

Conventional trial-error method is inefficient in discovering new functional materials in vast chemical and structural space. Here Lu et al. use machine learning techniques to screen out the most promising lead-free organic-inorganic perovskites with proper bandgap and stability from thousands of them in a flash.

## Introduction

The development of functional materials is the cornerstone of innovations in industry, and discovering materials with targeted property has always been a hotspot in science. The emergence of advanced techniques, such as high-throughput calculations based on density functional theory (DFT) has accelerated the search process at certain level^1–4^. However, the increasing scale of practical problems and complexity of materials require more sophisticated and effective methods for enormous database. Fortunately, the rapid development of material genome project^5^ and artificial intelligence technology has brought exciting hope to this dilemma^6–8^. Most recently, machine learning (ML) technology has been made significant progress in the rational material design, such as efficient molecular organic light-emitting diodes^9^, low thermal hysteresis shape memory alloys^10^, piezoelectrics with large electrostrains^11^ and so on. Bypassing complex quantum mechanics, ML technology can not only greatly accelerate materials design with high accuracy, but also learn trends within materials’ basic composition from big material data.

While ML technology sheds light on in the field of material design on inorganic perovskites^12–15^, the discovery of hybrid organic–inorganic perovskites (HOIPs) has never been reported yet in this way. HOIPs, as one of the most promising photovoltaic materials, have attracted tremendous interest recently. The most distinguished virtues of HOIPs includethe high power conversion efficiency (PCE), low-cost experimental synthesis and tunable bandgaps^16–20^. Since the first successful application of CH_3_NH_3_Pb*X*_3_ (*X* = Cl, Br) with a PCE of 3.8% in 2009 by Kojima et al.^21^, great efforts have continually been devoted to improve their PCE. Currently, the PCE of solar cells based on HOIPs has been boosted up to 22.1%^22^. Despite the great progress of HOIP-based solar cells, two key challenges limit the emerging materials for large scale commercial applications. One of the serious issues is toxicity, due to the element of lead (Pb), which contributes to most of the HOIP-based solar cells with high PCEs^23,24^. The other well-known factor is that their environmental stability is particularly poor, even with strict protection procedures. Therefore, it is of paramount importance to find stable Pb-free HOIPs with high PCE and sustainable air stability^25–28^. Unfortunately, due to the complexity and diversity of HOIPs structures (they are composed of organic molecules and inorganic metal frames), DFT-based high throughput calculations are too expensive and time consuming, not to mention experiments.

Here, we develop a target-driven method to discover stable Pb-free HOIPs based on ML technique and DFT calculations. We first train our ML model from 212 reported HOIPs’ bandgap values, and predict the bandgaps of 5158 unexplored possible HOIPs. A close structure-property relationship mapping HOIPs’ bandgap is concurrently excavated out from ML data, in which the ranges of tolerance factor, octahedral factor, metal electronegativity, and polarizability of organic molecules are defined for ideal HOIP-based solar cells. After further screening, six stable Pb-free HOIPs are selected as promising solar cells materials with proper bandgap.

## Results

### Design framework

**Fig. 1:**
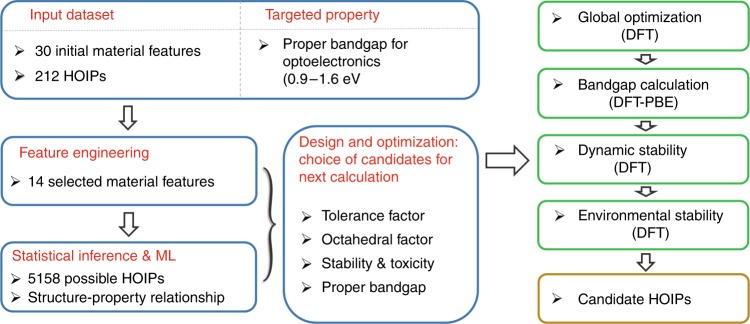
Lead-free HOIPs design framework. The material design framework combined with ML and DFT to efficiently search for stable Pb-free HOIPs with proper bandgap. The blue boxes represent the material screening process based on the ML algorithm generated from historical HOIP data. Then electronic properties and stability evaluation of these selected candidates are further calculated using DFT, which are shown in the green boxes

Our multi-stage material design approach is schematically illustrated in Fig. 1, and the prediction engine consists of three integral components: input HOIPs data, ML algorithm, and DFT calculations. As a common ML procedure, an input dataset of HOIPs, each of which is described by features, is built for training and testing ML model. With that, feature engineering is needed in the first place to remove redundant features and establish a structure-property relationship. Once the input feature set is fixed, the best hyper-parameters will be selected using grid searching technique and five-cross-validation procedures (the selection details are given in Methods)^29^. After that, we apply the trained ML model to the prediction dataset. Finally, DFT calculations are performed to study the thermal and environmental stabilities and electronic properties of HOIPs candidates screened out from ML simulations.

### Dataset

**Fig. 2:**
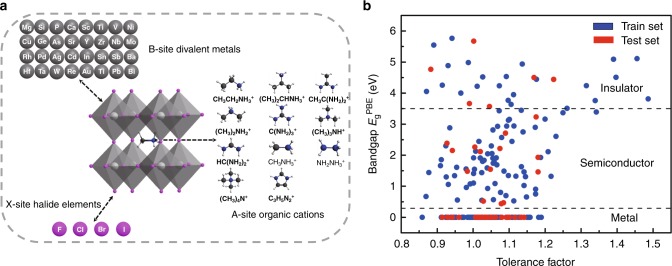
HOIPs input dataset for training and testing. **a** 212 high throughput HOIP structures. The combination of 11 small organic molecular species (A-site) and 32 divalent metals (B-site) constitutes the input samples of our ML model. X is a typical halide. **b** Data visualization in training (blue dots) and test (red dots) of tolerance factor and bandgap $E_{\mathrm{g}}^{\mathrm{PBE}}$ of HOIPs. The entire dataset consists of metals, semiconductors, and insulators

The input data for this study, containing 346 HOIPs, is obtained from previous high throughput first-principles calculations^30,31^. For data consistency and accuracy of ML predictions, we only select orthorhombic-like crystal structures with bandgap calculated using the Perdew-Burke-Ernzerh (PBE) functional. Therefore, 212 selected HOIP compounds are included in this engine, which completely belong to the family of perovskites of chemical formula AB*X*_3_, as explicitly shown in Fig. 2a. In this structure, the halogen atoms *X* (*X* = F, Cl, Br, I) occupy the vertices of regular corner-sharing BX_6_ octahedron, while 32 different divalent metal cations B^2+^ sit at the center of the octahedron, and 11 kinds of monovalent cations A^+^ fill in the cavity formed by the adjacent octahedrons. In the flow chart, we construct a dataset visualized in the form of plots between tolerance factor and bandgap $E_{\mathrm{g}}^{\mathrm{PBE}}$ of HOIPs, in which they are divided into a training dataset (80%) and a test dataset (20%) after one thousand test (see Fig. 2b and Supplementary Fig. 1). As we can see from the data distribution, the input HOIPs dataset is made up of three parts: metals (zero bandgap), semiconductors (bandgap between 0 and 3.5 eV) and insulators (bandgap larger than 3.5 eV).

In fact, there are plenty of choices for the sites A and B in a HOIP. For A-site, we collect other 21 kinds of organic molecular cations A^+^, all of which have been considered in the literature^20,32,33^ (see Supplementary Fig. 2). Meanwhile, we substitute the B-site with 43 divalent cations across the Periodic Table. Consequently, 5504 different possible HOIP compounds (32 A-site cations, 43 B-site cations and 4 X-site anions) are obtained. Considering that 346 HOIPs have been studied, we explore the rest 5158 potential candidates in this work.

### Feature engineering

For any ML method that targets toward a prespecified material property, it usually depends on a certain amount of features (descriptors). The features not only uniquely define each material in input dataset, but also relate to its desired physical and chemical properties. Although there may be many factors that affect the targeted property of materials, the number of features must be reasonable. The best strategy is to choose features that perfectly represent the materials’ property and the number of features should be far less than the number of materials in input dataset to avoid the curse of dimensionality^34^.

In this work, 30 initial features (the total features are in Supplementary Table. 1 and their sources are given in Supplementary Note. 1) such as ionic radii, tolerance factor and electronegativity are chosen to describe HOIPs in the chemical space collectively. In order to understand the relationship between features and targeted property, we evaluate the initial features via the gradient boosting regression (GBR) algorithm^35^. Furthermore, we incorporate a “last-place elimination” into the GBR algorithm to efficiently exclude the features that have less impact on the bandgap (the computational details are given in Methods). A detailed description of the feature selection procedure is given in and Supplementary Fig. 3 and Supplementary Note. 2. Finally, 14 most important features are sorted out and constitute as an optimal feature set. The new feature set contains structural features (tolerance factor *T*_f_, octahedral factor *O*_f_) as well as the elemental properties of A-, B- and X-site ions (total number of ionic charge IC_B_, p orbital electron *X*_p-electron_, ionization energy IE_B_, electronegativity *χ*_B_, electron affinity EA_B_, ionic polarizability (*P*_B_, *P*_A_), sum of the s and p orbital radii *r*_s+p B_, iron radii (*r*_B_, *r*_A_), the highest occupied molecular orbital and the lowest unoccupied molecular orbital of A site cations (HOMO_A_, LUMO_A_).

**Fig. 3:**
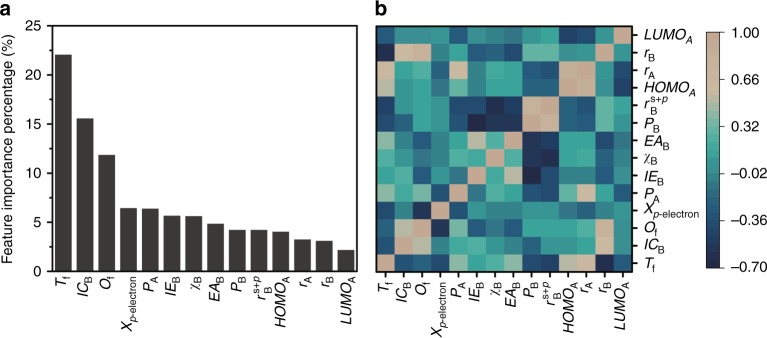
Importance and correlation of the selected features. **a** The 14 selected features are ranked using GBR algorithm. **b** The heat map of Pearson correlation coefficient matrix among the selected features for HOIPs

As shown in Fig. 3a, the tolerance factor *T*_f_ plays the most important role to HOIPs’ bandgap, followed by the total number of ionic charge IC_B_ and the octahedral factor *O*_f_. Interestingly, the properties of B-site elements such as ionization energy IE_B_, electronegativity *χ*_B_ and electron affinity EA_B_ show greater influence on the bandgap of HOIPs than those of A- and X-site ions. Pearson correlation coefficient matrices are calculated to identify the positive and negative correlations between pairs of features (Fig. 3b). The low linear correlations for most of features indicate that we have successfully removed redundant and irrelevant features, which will significantly improve the performance of the ML model.

### Model inference

In ML method, an appropriate ML algorithm is important. Currently, several supervised ML regression algorithms have been successfully used in material science, such as GBR^32^, artificial neural network^36,37^, and kernel ridge regression (KRR)^38^. These regression algorithms provide both material property prediction with DFT accuracy and atomic level chemical insights. In this work, we employ six different ML regression algorithms, i.e., GBR, KRR, support vector regression, gaussian process regression, decision trees regression, and multilayer perceptron regression. Each training model is based on a subset of the whole data, known as training data, and the model will be used to predict other new data after training. To evaluate the performance of each ML model, three indexes are chosen to estimate the prediction errors: coefficient of determination (*R*^2^), Pearson coefficient (*r*), and mean square error (MSE) (the computational details are given in Supplementary Methods). By comparing the three indexes, GBR algorithm reproduces best agreement to the true bandgap values (see details in Supplementary Fig. 4 and 5). Then, we perform a statistical test on *R*^2^ values from 10,000 executions of each model at the 95% confidence level (see details in Supplementary Table. 2 and 3). Evident differences are observed between GBR and other five algorithms. Furthermore, we put standard deviations on the *R*^2^ and MSE values. It is found that GBR algorithm presents more reliable results than other five algorithms (see details in Supplementary Fig. 6 and Supplementary Note. 3). Additionally, the GBR algorithm evolves from the combination of boosting methods and regression trees, which makes it suitable for effectively mining features and feature engineering^39^. Therefore, GBR is chosen to establish a nonlinear mapping between the input features and bandgaps and subsequently predicts bandgaps of unexplored HOIPs.

**Fig. 4:**
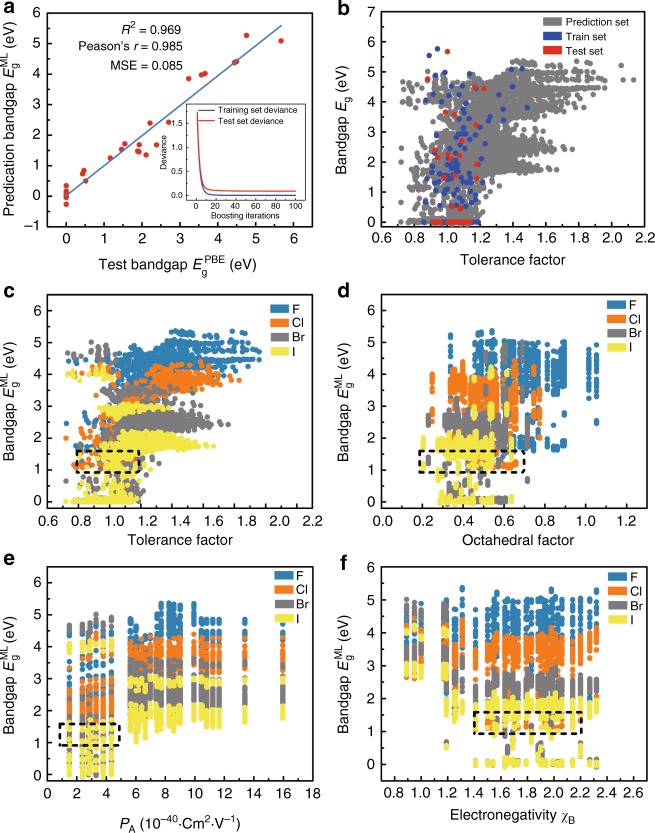
Results and insights from ML model. **a** The fitting results of test bandgaps $E_{\mathrm{g}}^{\mathrm{PBE}}$ and predicted bandgaps $E_{\mathrm{g}}^{\mathrm{ML}}$. Coefficient of determination (*R*^2^), Pearson coefficient (*r*) and mean squared error (MSE) are computed to estimate the prediction errors. The subplot is the convergence of model accuracy for five cross-validation split of the data. **b** Scatter plots of tolerance factors against the bandgaps for the prediction dataset from trained ML model (blue, red and dark gray plots represent train, test and prediction set, respectively). Data visualization of predicted bandgaps for all possible HOIPs (one color represents a class of halogen perovskites) with (**c**) tolerance factor, (**d**) octahedral factor, (**e**) ionic polarizability for the A-site ions, and (**f**) electronegativity of B-site ions. The dotted box represents the most appropriate range for each feature

The test results of the GBR model are presented in Fig. 4a. The subplot clearly shows that the training/test set deviance declines gradually with the increase of the boosting iteration numbers. Eventually, *R*^2^, *r,* and MSE of test data is 97.0%, 98.5%, and 0.086, respectively, witnessing the outstanding performance of our GBR model. Then, the trained GBR model is applied to the 5158 HOIPs to predict their bandgaps, and the prediction results (dark gray dots) are illustrated in Fig. 4b, together with train dataset (blue dots) and test dataset (red dots). We notice that the distribution of post predicted bandgaps is very close to the original input dataset. This proves the reasonability and reliability of our ML model, providing guarantee for further analysis.

Furthermore, data analysis and visualization are performed to unravel hidden trends and periodicities within the HOIPs data. We divide the predicted dataset into four parts according to the X-site elements, i.e., F, Cl, Br, and I in Fig. 4c-f. As seen from the figure, the bandgap of HOIPs tends to increase as the X-site halogen radius reduces and the bandgaps of F- and Cl- HOIPs are too large for photovoltaics applications. Therefore, HOIPs containing Br and I (ABBr_3_ and ABI_3_) with promising prospect for photovoltaic applications are mainly focused in the following discussion. Considering the structural stability for HOIPs, the given features should be restricted in certain ranges. Specifically, tolerance factor *T*_f_, which has been used extensively to predict the stability of the perovskite structure, should be between 0.8 and 1.2 (Fig. 4c). In terms of the octahedral factor *O*_f_, the appropriate range for solar cells is between 0.2 and 0.7 (Fig. 4d). However, the octahedral factor should not be too small for structural stability, so the values are better ranging from 0.4 to 0.7^33,40,41^. Moreover, in order to design HOIPs with proper bandgap, we should select weak polarized organic molecules and the polarization *P*_A_ varies between 1 × 10^−40^ and 5 × 10^−40^ C m^2^ V^−1^ (see Fig. 4e). Finally, the electronegativity of B-site also plays important roles on the bandgap and it needs to be within the range from 1.4 to 2.2 (Fig. 4f). We also analyze the mapping between other eight features with the bandgaps of HOIPs and less obvious correlations are observed (see Supplementary Fig. 7).

### Model validation

We have predicted bandgaps *E*_g_ for all the possible HOIP structures in the search space via ML technology. To discover stable HOIPs, further screening of the predicted dataset is necessary. 1669 HOIPs are first screened out from the total 5158 HOIPs with ML predicted bandgaps according to the structural stability (*T*_f_ between 0.8 and 1.2, *O*_f_ between 0.4 and 0.7). These 1669 HOIPs are likely stable and have different potential applications in light of their bandgap values. For examples, HOIPs with small bandgaps (less than 0.9 eV) can be used in infrared sensors^42,43^ and large gap HOIPs (larger than 3 eV) may serve as good insulating materials^44,45^. For solar cells, HOIPs with bandgap between 0.9 and 1.6 eV are ideal candidates^46,47^. Therefore, 218 HOIPs with proper bandgap are selected (see full list in Supplementary Table. 4). Since the Br-based HOIPs are more accessible in experiment^19^, 22 Br-based HOIPs (i.e., ABBr_3_) are further selected. Additionally, toxicity of HOIPs will block widespread commercial application and the compounds containing toxic metal elements are excluded as well. To the end, six orthorhombic HOIPs stand out and further thermal and environmental stability evaluation and electronic property exploration are performed by first-principle calculations. The step-by-step screening process are shown in Supplementary Fig. 8 and Supplementary Note 4.

**Fig. 5:**
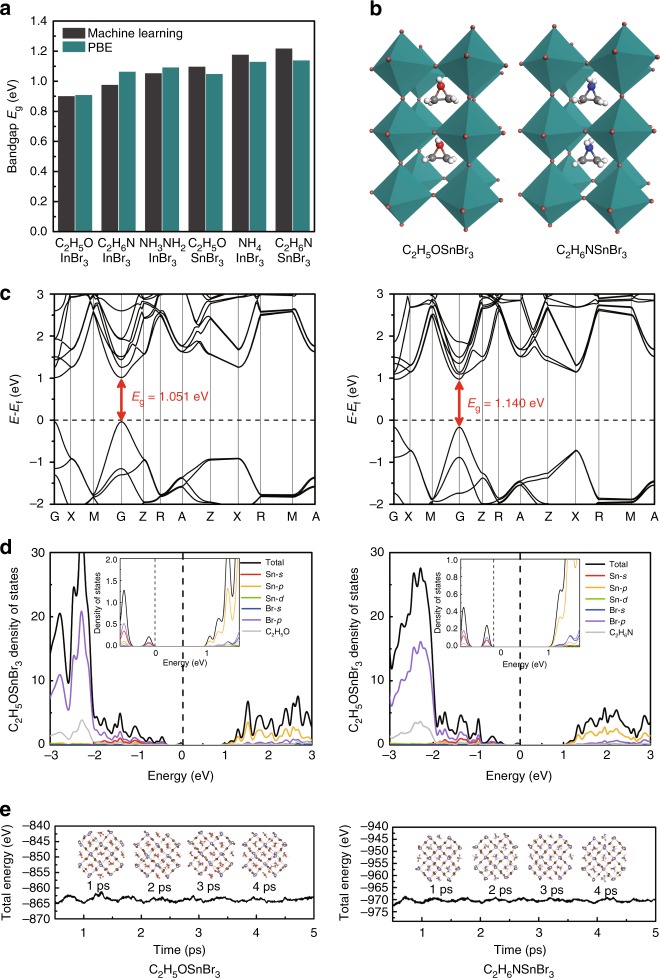
Comparison with DFT calculations. **a** A comparison between ML-predicted and DFT-calculated results of six selected HOIPs. **b** Optimized structures, **c** band structures, **d** projected density of states (PDOS), **e** total energy during 5 ps AIMD simulations for C_2_H_5_OSnBr_3_ and C_2_H_6_NSnBr_3_ at 300 K. The subplots in **d** are the PDOS near the Fermi level

A comparison between ML-predicted and DFT-calculated results of six selected HOIPs is presented in Fig. 5a, with relevant statistics summarized in Table 2. Excellent agreement (Δ*E*_g_ no larger than 0.1 eV) is found between the ML predicted and DFT calculated bandgap values, verifying the great superiority of the current ML technology. It takes only a few seconds for all the 5158 HOIPs’ bandgaps to be predicted by the ML method. However, if the DFT calculation is adopted, it will take a few days for each HOIP structure. Therefore, we conclude that our current ML scheme provides a possibility of achieving DFT accuracy in a flash and has great priority in complex systems like HOIPs.

### Electronic structures of six selected HOIPs

DFT optimizations show that all these six HOIPs hold typical perovskite structures, and corresponding lattice constants are listed in Table 1. The nonbonding electrons of B-site ions lower the coordination symmetry around the cations and lead to the slightly distorted BX_6_ octahedral. Sorted by B site metal, the six candidates can be divided into two groups: four AInBr_3_ and two ASnBr_3_. Further electronic band structures show that the four indium HOIPs have indirect bandgap between Γ and M/R point in the Brillouin zone (Supplementary Fig. 9 and 10), while the other two tin HOIPs have direct bandgap at Γ point (Fig. 5c). Additionally, the spin-orbital-coupling (SOC) effect is considered for the six selected HOIPs (see details in Supplementary Fig. 11 and Supplementary Note 5) and the influence to the band structure is not pronounced.

**Table 1:** Lattice constants of six selected HOIPs

HOIPs	*a* (Å)	*b* (Å)	*c* (Å)	*α* (°)	*β* (°)	*γ* (°)
C_2_H_5_OInBr_3_	8.44	11.46	8.48	91.63	91.76	91.58
C_2_H_6_NInBr_3_	8.50	11.7	8.12	92.17	90.35	88.52
NH_3_NH_2_InBr_3_	8.33	11.68	7.67	88.71	89.73	88.69
C_2_H_5_OSnBr_3_	8.52	11.60	8.52	91.32	89.74	89.88
NH_4_InBr_3_	7.92	11.53	7.88	90.42	90.00	90.43
C_2_H_6_NSnBr_3_	8.63	11.95	8.26	91.78	89.42	88.58

*a*, *b*, and *c* are lattice length. *α*, *β,* and *γ* are lattice angle

As typical electronic structures of ASnBr_3_ and AInBr_3_, the valence band maximums are mainly contributed by the p orbital of the halogen atoms and partly contributed by the s orbital of the metal atom, while the conduction band minimums are dominated by p orbitals of the metal atom (Fig. 5d and Supplementary Fig. 9 and 10). In fact, as a result of the indirect bandgap of AInBr_3_, the absorption coefficient will be relatively low. Therefore, a relatively thick absorbent layer is required, which definitely increases the material costs, especially with the high cost of rare metal indium. Hence, two ASnBr_3_ HOIPs are better choices as light-harvesting materials in photovoltaic devices with suitable direct bandgaps and relatively low material costs.

### Thermal and environmental stabilities of six selected HOIPs

The thermal and environmental stabilities are important to the practical application of HOIPs, and most reported structures with high PCEs suffer from degradation in ambient. We first perform ab initio molecular dynamics (AIMD) simulations to evaluate the thermal stability of the six selected HOIPs. As shown in Fig. 5e and Supplementary Fig. 9 and 10, the time-dependent evolutions of total energies are oscillating within a very narrow range, indicating that these HOIPs can maintain their structural integrity at room temperature. In the next step of evaluating the environmental stabilities, the adsorption energies (Δ*E*_ads_) of molecular water and oxygen on (001) surfaces of these six HOIPs are calculated and listed in Table 2 (the computational details can be found in Supplementary Fig. 12). Comparing with MAPbI_3_ whose adsorption energies of water and oxygen are −0.48 and −0.15 eV, C_2_H_5_OInBr_3_, C_2_H_5_OSnBr_3_, and C_2_H_6_NSnBr_3_ show better environmental stability against both oxygen and water. Although the degradation mechanism of HIPO is still under discussion, it is widely accepted that water acts as the reactive source for the collapse of the framework. We attribute the significant reduction in adsorption energy of the three systems for water to the weaker polarity of C_2_H_5_O^+^ and C_2_H_6_N^+^ comparing with MA^+^ radical, which present strong interaction with water through hydrogen bond. Therefore, the weak hygroscopicity of C_2_H_5_OInBr_3_, C_2_H_5_OSnBr_3_, and C_2_H_6_NSnBr_3_ suggests that the H_2_O molecules are reductant to aggregate on the surface, and increase the energy barrier of the H_2_O penetration process in the meantime, preventing the hydration degradation effectively.

**Table 2:** Six selected HOIPs with relevant statistics

HOIPs	*T*_f_	*O*_f_	*E*^*ML*^_g_(eV)	*E*_g_^PBE^(eV)	$\Delta{{E}_{{\bf{H}}_{\bf 2 }\bf{O}}}_{\bf ads}$(${\bf eV}$)	$\Delta{{E}_{{\bf{O}}_{\bf 2}}}_{\bf{ads}}$(${\bf eV}$)
C_2_H_5_OInBr_3_	1.04	0.50	0.90	0.91	−0.301	−0.071
C_2_H_6_NInBr_3_	1.04	0.50	0.97	1.07	−0.630	−0.152
NH_3_NH_2_InBr_3_	0.99	0.50	1.06	1.09	−0.497	−0.110
C_2_H_5_OSnBr_3_	0.99	0.57	1.10	1.05	−0.163	−0.071
NH_4_InBr_3_	0.82	0.50	1.18	1.13	−0.566	−0.090
C_2_H_6_NSnBr_3_	0.99	0.57	1.22	1.14	−0.134	−0.093

*T*_f_ and *O*_f_ is tolerance factor and octahedral factor respectively. *E*_g_^ML^and *E*_g_^PBE^ are ML-predicted and DFT-calculated results respectively. Δ*E*_g_ is the absolute value of the difference between *E*_g_^ML^ and *E*_g_^PBE^. Δ*E*_H_2O ads and Δ*E*_O_2 ads is the adsorption energy of H_2_O and O_2_, respectively

## Discussion

Combining with ML technology and DFT calculations, we have developed an extremely fast target-driven method to discover HOIPs. Three stable Pb-free HOIPs with proper bandgaps and excellent thermal and environmental stabilities have been successfully selected out from 5158 HIOPs for solar cells. More importantly, a close structure-property map of HOIPs has been well established from the ML predicted big dataset and four stringent conditions in terms of tolerance factor, octahedral factor, electronegativity of metal ions, and polarizability of organic molecules are defined to be ideal HOIP-based solar cells.

Differently from those high-throughput screening methods which the whole chemical space should be searched at DFT level, the current ML and DFT combined scheme only needs to compute the most promising HOIPs at DFT level, which greatly saves the computational resources. Note that the screenings described above are very strict, and in fact, the screening conditions can be adjusted according to targeted goal to find suitable candidates for experimental synthesis. The target-driven method we developed here overcomes a major obstruction in traditional trial-error method. Meanwhile, as this ML technology employs a “last-place elimination” feature selection procedure based on GBR algorithm, it can not only achieve DFT accuracy in a flash (even faster than the popular neural network algorithm), but also works with a small dataset. This means we can achieve accurate prediction with a relatively small training data. Here we only apply this intelligent method to accurately predict the bandgaps of thousands of HOIPs and get over the problem of toxicity and poor environmental stability in HOIPs. In fact, it is applicable to other functional material design and discovery, if the computational or experimental material data are enough to train the ML model.

## Methods

### Gradient boosted regression

GBR^35,48^, a flexible non-parametric statistical machine leaning algorithm in the open-source *scikit-learn* package^49^, is implemented to predict bandgaps of undiscovered HOIPs. The learning principle of this method is to improve the accuracy of the final regression results by gradually reducing the algorithm generated by the training process. The final regression algorithm is the weighted sum of several weak regression algorithms obtained by each training, as

1 $$F_M\left( x \right) = \mathop {\sum}\limits_{m = 1}^M {T\left( {x,\theta _m} \right)},$$

where *m* is the times of training, *x* is the input data, and *θ*_*m*_ is the distribution weight vector. The model is trained *M* times, and each time it produces a weak regression function *T*. The loss function of every weak classifier, is defined as

2 $$\hat \theta _{m} = \mathop{\arg \hskip 2pt \min}\limits_{\theta _m}\mathop {\sum}\limits_{i = 1}^{N} L \left({y_{i},F_{m - 1}\left( {x_i} \right) + T\left( {x_{i},\theta _{m}} \right)} \right),$$

where *F*_*m*-1_(*x*_*i*_) is the current model, and GBR determines the parameters of the next weak classifier through empirical risk minimization. This work uses ML to analyze a small dataset based on DFT calculation to construct a predictive model.

### Hyper-parameters selection

In the ML algorithm, the hyper-parameter is the parameter set before the learning process, rather than that obtained through training model. In general, it is necessary to select a set of optimal hyper-parameters for the learning machine to improve the efficiency and generalization performance of the model. Here, six hyper-parameters in the GBR model are optimized by grid searching method: loss function (least squares), learning rate (0.2), maximum depth of the individual regression estimators (12), the number of features to consider when looking for the best split (0.7), the minimum number of samples required to be at a leaf node (3) and the number of boosting stages to perform (100). The values in parentheses represent the best results for each hyper-parameter.

### Last-place elimination feature selection procedure

We employ a “last-place elimination” feature selection procedure into GBR algorithm to optimize the most relevant features. Thirty initial features, which are commonly employed in ML algorithm or structure features of HOIPs, are considered and first ranked by GBR algorithm according to the relative importance. Then we remove the least important feature (i.e., the 30th feature) out of the feature set and the rest 29 features constitute a new dataset for the next step feature selection. After that, we rank the remaining features again and repeat the above step. Finally, we train the ML model using different datasets for twenty-nine times (see workflow in Supplementary Fig. 3). We record the model score (*R*^2^) of each trained model and the ML model performs best when the feature set only includes fourteen features.

### Density functional theory

All DFT calculations for selected HOIPs are carried out using the projector-augmented wave method with the generalized gradient approximation, implemented in the Vienna Ab initio Simulation Package package^50^. The exchange-correlation functional is described by PBE^51^ functional considering it reproduces more consistent results with the experiments for HOIPs due to fortuitous error–error offset^52,53^. DFT-D3 method is adopted for the van der Waals correction^54^. AIMD simulations are performed at room temperature by using the Nosé-Hoover method^55,56^ to verify the thermal stability of selected materials. The environmental stability of selected HOIPs are further evaluated by the adsorption energy calculations. More DFT calculation details can be found in Supplementary Methods.

### Data availability

The datasets generated during and/or analyzed during the current study are available from the corresponding author on reasonable request.

## Electronic supplementary material

Supplementary Information File
