# Supplementary material for: Accelerated discovery of stable lead-free hybrid organic-inorganic perovskites via machine learning
Source: Nat Commun. 2018 Aug 24;9:3405. doi: 10.1038/s41467-018-05761-w (PMC6109147; doi:10.1038/s41467-018-05761-w)
Supplement: Supplementary file 1 — Supplementary Information File [file 41467_2018_5761_MOESM1_ESM.pdf]

Accelerated discovery of stable lead-free hybrid organic-inorganic perovskites via machine learning

Shuaihua Lu[†], Qionghua Zhou[†], Yixin Ouyang, Yilv Guo, Qiang Li and Jinlan Wang[*]

School of Physics, Southeast University, Nanjing, 211189, China

Supplementary Figures

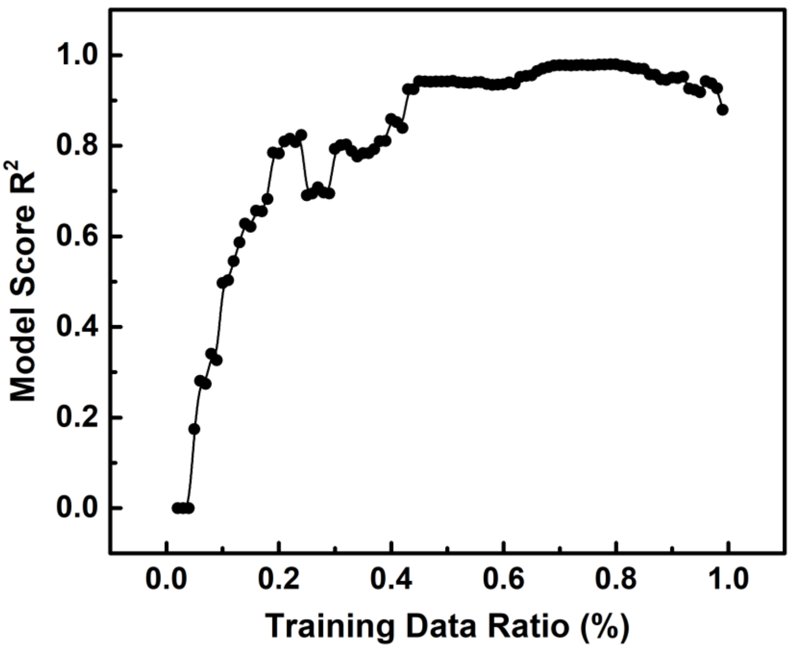

Supplementary Figure 1 | Training dataset ratio selection. The test percentage of training data set is from 1% to 99%. Each training data set is used to train the ML model and record the model score R^2. As is shown in Supplementary Fig. 1, when the training data radio is up to 80%, the ML model performs best. So we split the input data set in to training dataset (80%) and test dataset (20%).

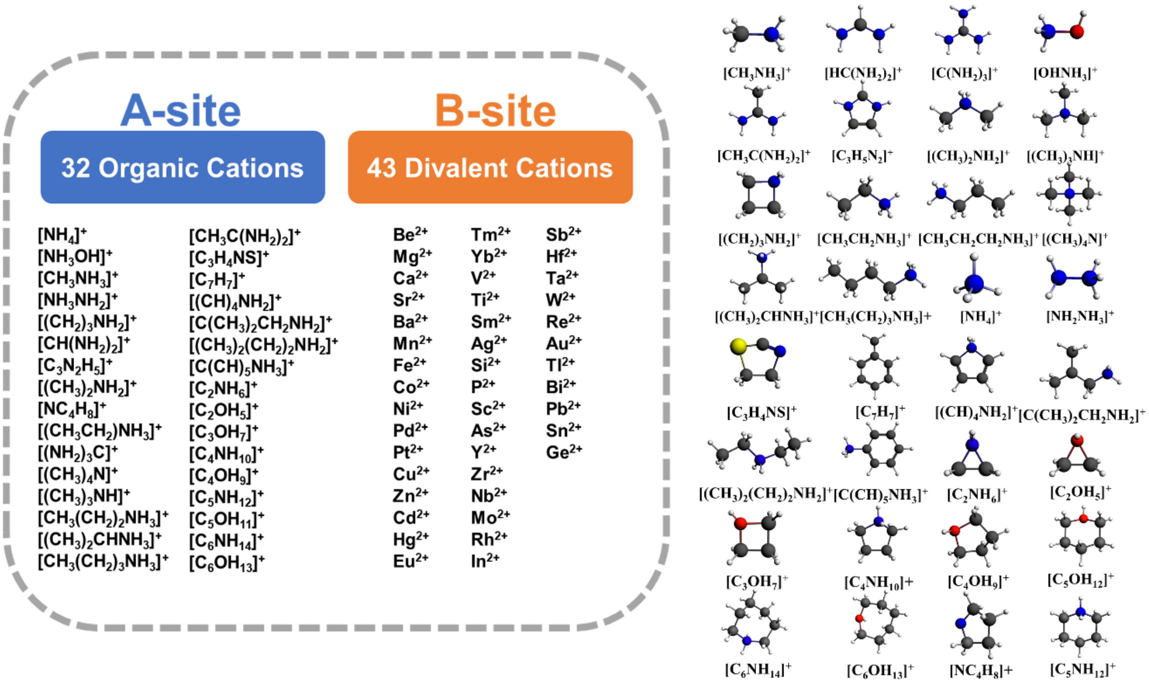

Supplementary Figure 2 | A- and B-site cations in prediction dataset. Another 21 organic molecules are collected as potential cations A^+, all of which have been considered in the literature. Simultaneously, we substitute the B-site with 43 divalent cations across the Periodic Table. Finally, the 32 organic cations and 43 divalent cations are represented in Supplementary Fig. 2, which lead to 5158 new HOIPs components.

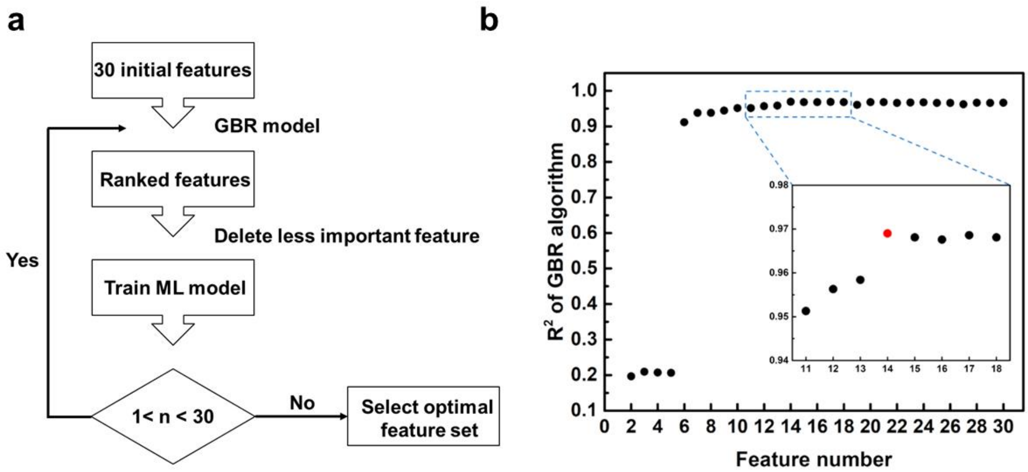

Supplementary Figure 3 | The feature selection procedure. (a) The 'last-place elimination' workflow. **(b)** R^2 of GBR model in each selection process. The blue line is polynomial fit of R^2. The position of the dotted line is the maximum value of R^2.

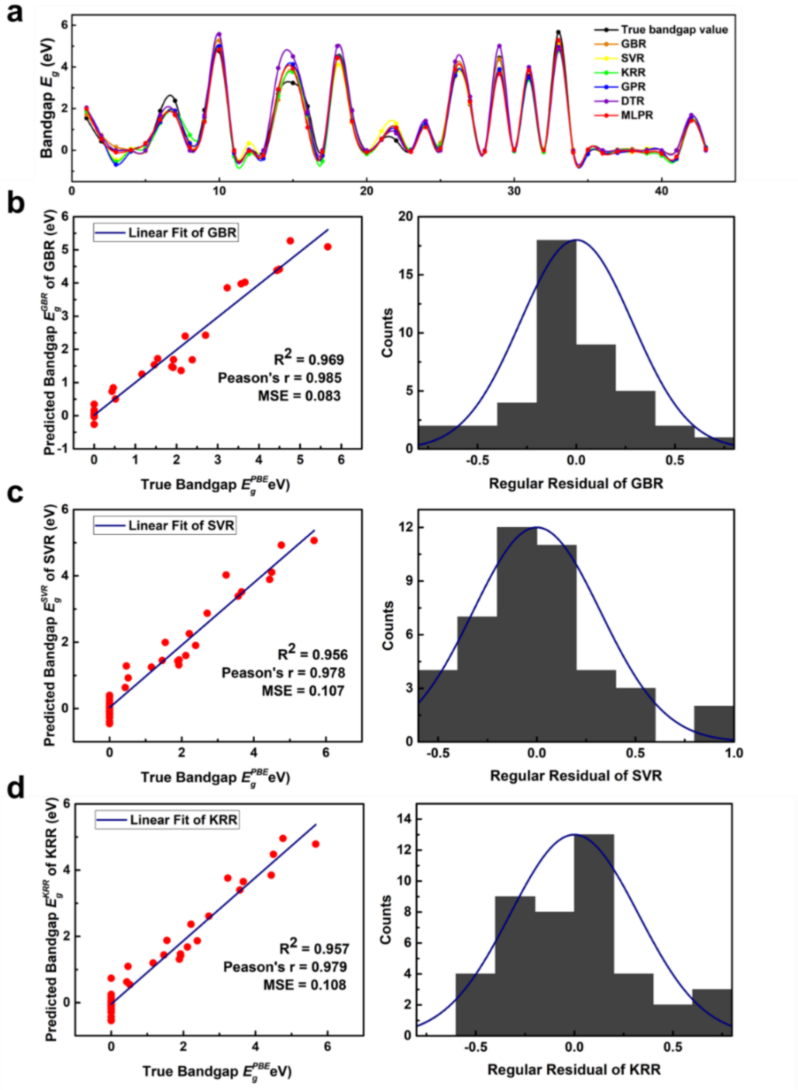

Supplementary Figure 4 | Learning performances of ML models. (a) Predicted bandgap values by six ML models for HOIPs, respectively. Each point is the average predicted value over ten thousand executions of each ML model on the test dataset of HOIPs. The test data are obtained via hold-out method, which cause all of them are not in the training dataset. The curves show the smoothed predictions. Scatter plots of true bandgap values E_g^{PBE} against predicted bandgap values E_g^{ML} by **(b)** gradient boosting regression (GBR) model, **(c)** support vector regression (SVR) model[1] and **(d)** kernel ridge regression (KRR) model[2]. The coefficient of determinations (R^2), Pearson correlation coefficient (r), mean squared error (MSE) and the counts of regular residual for each ML model are represented, showing learning performances of each ML model.

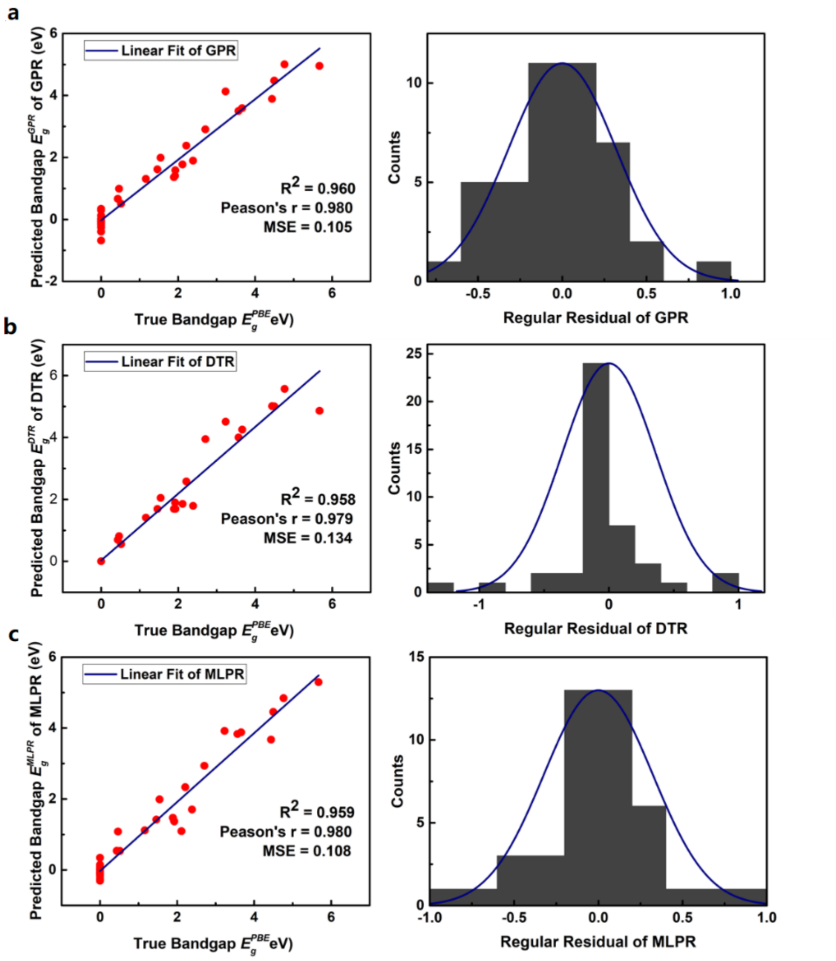

Supplementary Figure 5 | Learning performances of ML models. Scatter plots of true bandgap values E_g^{PBE} against predicted bandgap values E_g^{ML} by **(a)** gaussian process regression (GPR) model[3], **(b)** decision trees regression (DTR) model[4] and **(c)** multi-layer perceptron regression (MLPR) model[5,6] are illustrated. The coefficient of determinations (R^2), Pearson correlation coefficient (r), mean squared error (MSE) and the counts of regular residual for each ML model are represented, showing learning performances of each ML model.

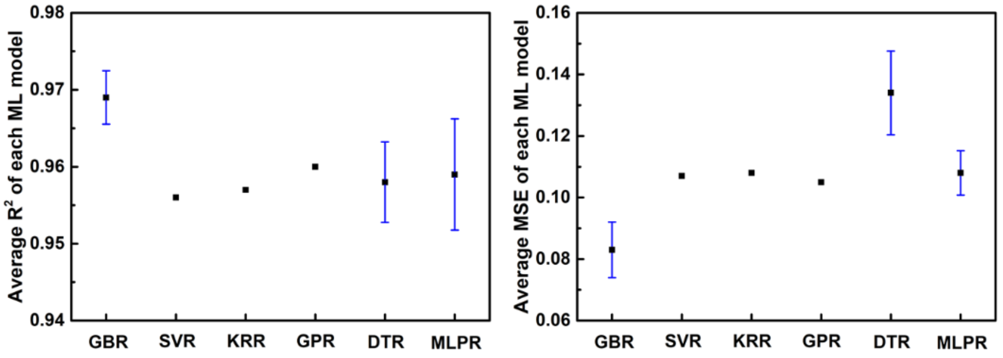

Supplementary Figure 6 | Average r^2 and MSE values of six ML models with standard deviations

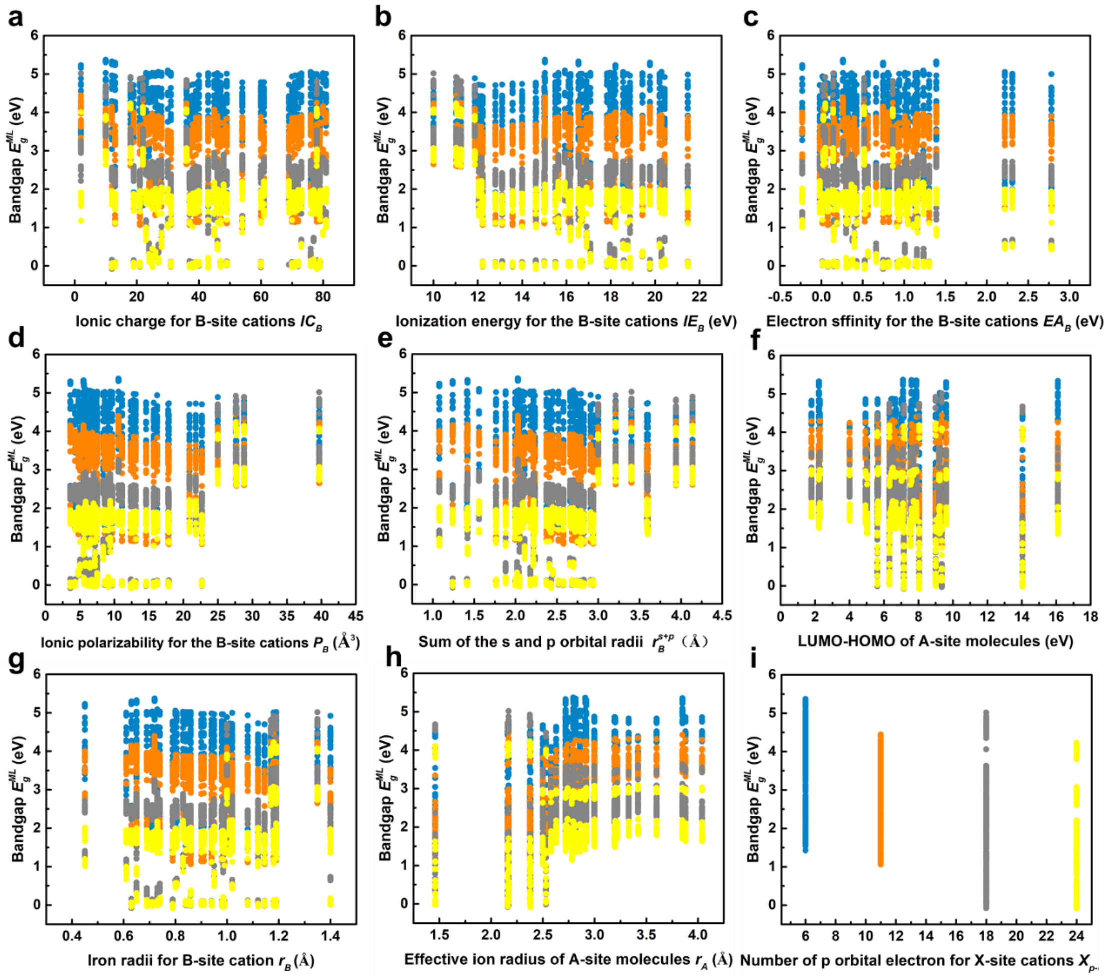

Supplementary Figure 7 | The structure-property relationship between HOIPs bandgap and features. **(a)** Ironic charge for B-site cations IC_B, **(b)** Ionization energy for the B-site cations IE_B, **(c)** Electron affinity for the B-site cations EA_B, **(d)** Ionic polarizability for the B-site cations P_B, **(e)** Sum of the *s* and *p* orbital radii r_B^{s+p} , **(f)** LUMO-HOMO of A-site molecules, **(g)** Iron radii for B-site cations r_B, **(h)** Effective ion radius of A-site molecules r_A and **(i)** Number of p orbital electron for X-site cation $X_{p\text{-electron}}$.

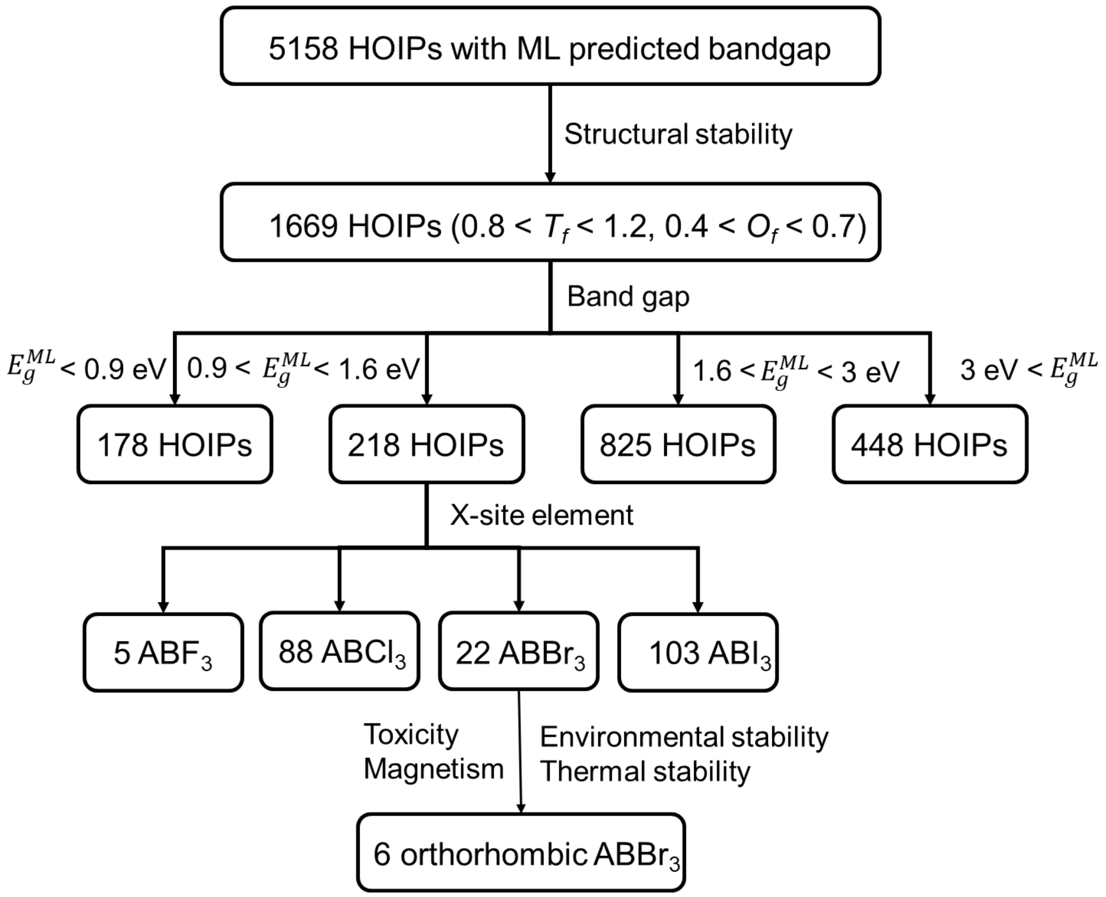

Supplementary Figure 8 | Optimal HOIPs screen workflow.

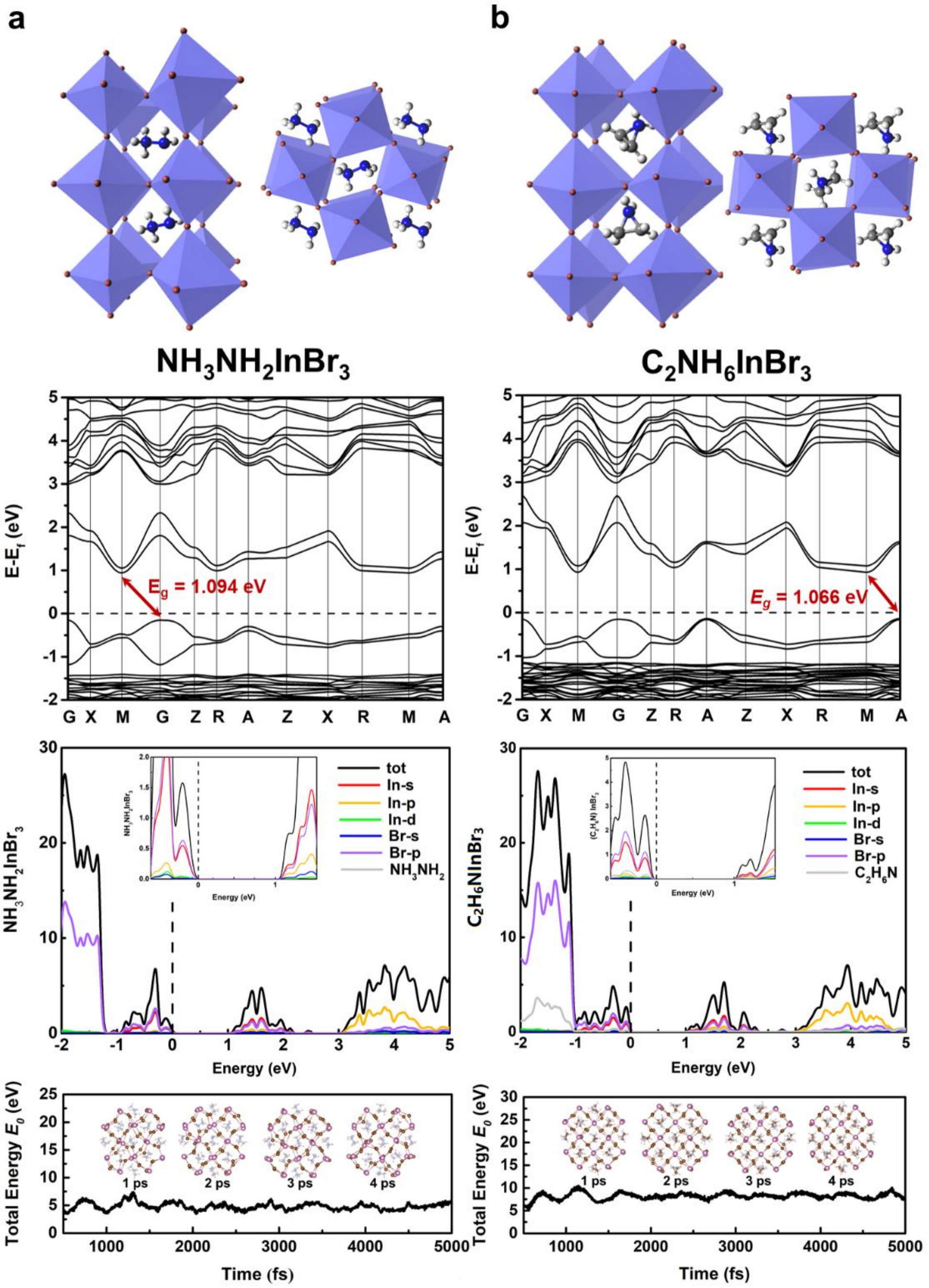

Supplementary Figure 9 | DFT calculation results for $NH_3NH_2InBr_3$ and $C_2H_6NInBr_3$. The optimized structures, band structures, PDOS and total energy during 5 ps AIMD simulations for **(a)** $NH_3NH_2InBr_3$ and **(b)** $C_2H_6NInBr_3$. The AIMD simulated results show that the time-dependent evolutions of total energies are oscillating within a very narrow range, indicating that these HOIPs can maintain their structural integrity at room temperature.

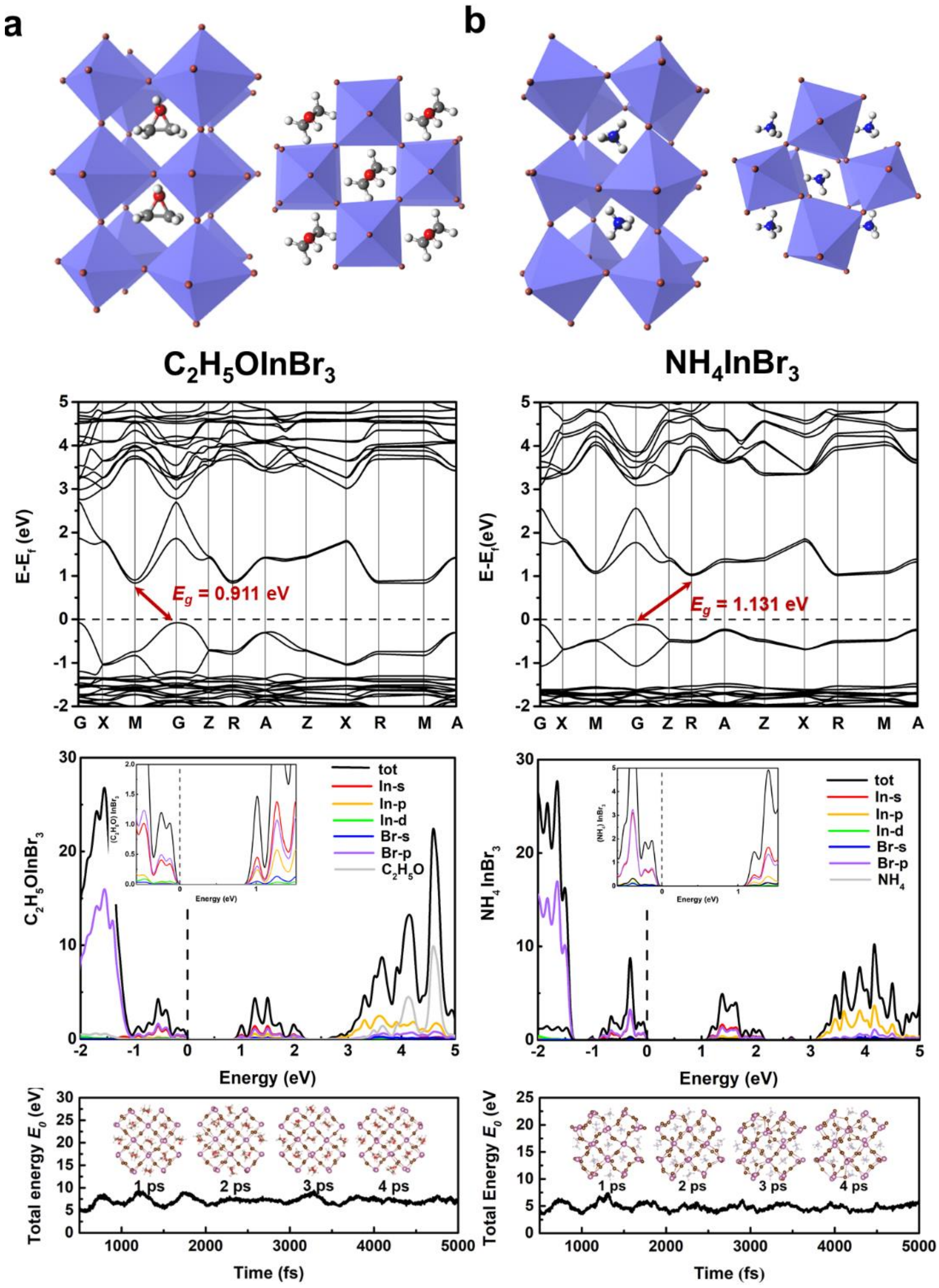

Supplementary Figure 10 | DFT calculation results for $C_2H_5OInBr_3$ and NH_4INBr_3. The optimized structures, band structures, PDOS and total energy during 5 ps AIMD simulations for **(a)** $C_2H_5OInBr_3$ and **(b)** NH_4InBr_3. The AIMD simulated results show that the time-dependent evolutions of total energies are oscillating within a very narrow range, indicating that these HOIPs can maintain their structural integrity at room temperature.

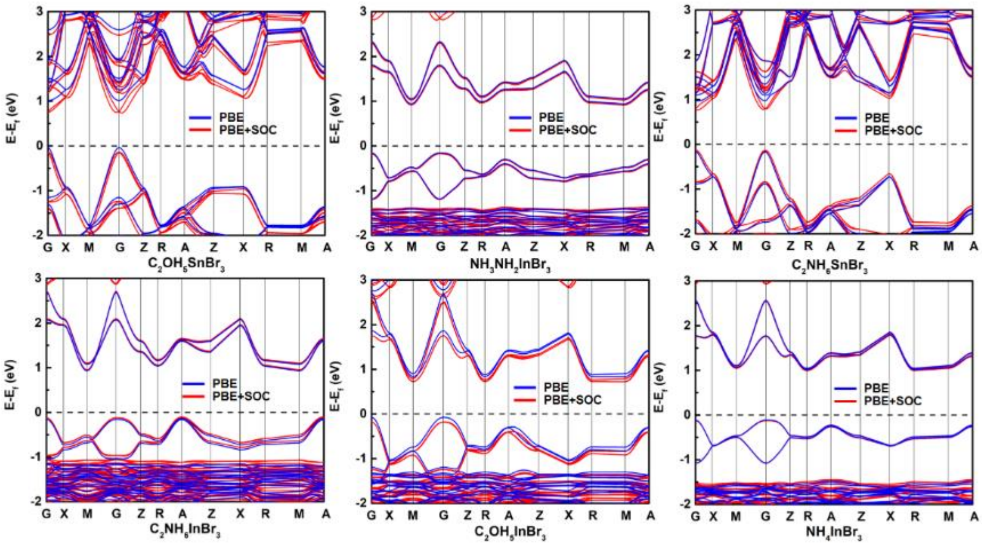

Supplementary Figure 11 | Band structure of six selected HOIPs. The band structures are calculated at PBE (blue line) and PBE+SOC (red line) level.

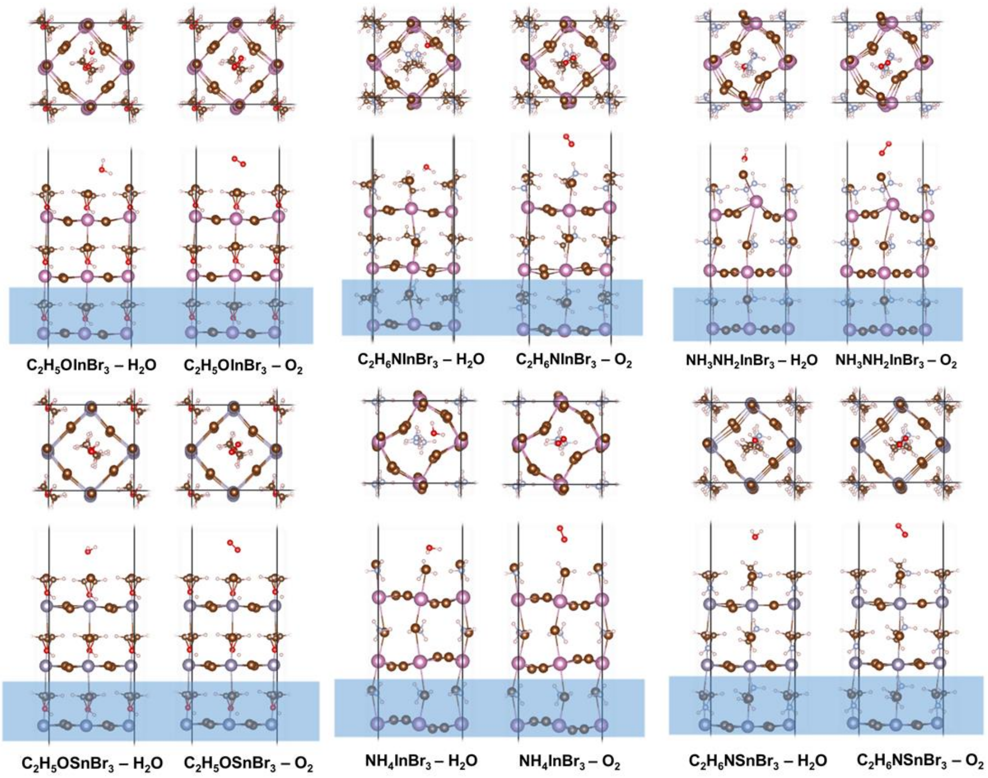

Supplementary Figure 12 | Structure of HOIPs with one H_2O/O_2 adsorbed on after optimization. Top and side views of six selected HOIPs' (001) surfaces containing water and oxygen after optimization, where atoms are fixed in the blue region for DFT calculation.

Supplementary Tables

Supplementary Table 1 | Thirty initial features with description.

Features	Description
$r^i_{\mathrm{A,eff}}$, r^i_{B}, and r^i_{X}	Iron radii for the A-, B- and X-site atoms [7-9]
T_f	Tolerance factor defined as $\frac{r^i_{\mathrm{A,eff}}+r^i_{\mathrm{X}}}{\sqrt{2}(r^i_{\mathrm{B}}+r^i_{\mathrm{X}})}$ [10, 11]
O_f	Octahedral factor defined as $\frac{r^i_{\mathrm{B}}}{r^i_{\mathrm{X}}}$ [12]
χ_{B}, χ_{X}	Martynov-Batsanov electronegativity scales [13, 14]
r^{s+p}_{B}, and r^{s+p}_{X}	Sum of the *s* and *p* orbital radii [15]
$B_{\text{x-electron}}$ (x = s, *p*, *d*, *f*)<br>$X_{\text{x-electron}}$ (x = s, *p*, *d*, *f*)	Numbers of *s*, *p*, *d* and *f* orbital electron for the B- and X-site cations
P_{A}, P_{B}, P_{X}	Ionic polarizability for the A-, B- and X-site ionic [16]
$\mathrm{HOMO_A}$, $\mathrm{LUMO_A}$	HOMO and LUMO for the A-site molecules
$\mathrm{IE_B}$	Ionization energy for the B-site cations [17]
$\mathrm{EA_B}$	Electron affinity for the B-site atoms [18]
$1^{\mathrm{st}}IP_{\mathrm{B}}$, $1^{\mathrm{st}}IP_{\mathrm{X}}$	The first ionization energy for B- and X-site atoms
$\mathrm{IC_B}$, $\mathrm{IC_X}$	Ionic charge for B- and X-site cations
$\mathrm{VE_B}$	Valence electrons for the B-site atoms

**Supplementary Table 2 | Test of Normality**	
Paired Samples	Kolmogorov-Smirnov[a]
	Sig.
GBR-SVR	0.000
GBR-KRR	0.000
GBR-GPR	0.000
GBR-DTR	0.000
GBR-MLPR	0.000
a. Lilliefors means significant level correction	

Supplementary Table 3 | Paired Samples *t* Test					
Paired Samples	Paired Differences			*t*	Sig. (2-tailed)
	Mean	95% Confidence Interval of the Difference			
		Lower	Upper		
GBR - SVR	0.012	0.01168	0.01181	338.862	0.000
GBR - KRR	0.013	0.01327	0.01341	385.024	0.000
GBR - GPR	0.008	0.00828	0.00841	240.768	0.000
GBR - DTR	0.032	0.03148	0.03173	502.676	0.000
GBR - MLPR	0.015	0.01495	0.01527	189.088	0.000

Supplementary Table 4 | 218 HOIPs with predicted bandgap E_g^{GBR} between 0.9 eV and 1.6 eV

A	B	X	E_g^{GBR} (eV)	A	B	X	E_g^{GBR} (eV)
NH_4^+	V	F	1.43	$NH_3NH_2^+$	Hg	Cl	1.58
NH_4^+	Ti	F	1.57	$NH_3NH_2^+$	V	Cl	1.28
NH_4^+	Rh	F	1.55	$NH_3NH_2^+$	Ti	Cl	1.10
NH_4^+	Re	F	1.60	$NH_3NH_2^+$	Ag	Cl	1.17
NH_3OH^+	Ti	F	1.57	$NH_3NH_2^+$	Sc	Cl	1.10
NH_4^+	Pd	Cl	1.33	$NH_3NH_2^+$	Y	Cl	1.10
NH_4^+	Pt	Cl	1.52	$NH_3NH_2^+$	Zr	Cl	1.10
NH_4^+	Hg	Cl	1.53	$NH_3NH_2^+$	Nb	Cl	1.10
NH_4^+	V	Cl	1.15	$NH_3NH_2^+$	Mo	Cl	1.17
NH_4^+	Ti	Cl	1.13	$NH_3NH_2^+$	Rh	Cl	1.17
NH_4^+	Ag	Cl	1.19	$NH_3NH_2^+$	Sb	Cl	1.22
NH_4^+	Sc	Cl	1.14	$NH_3NH_2^+$	Hf	Cl	1.18
NH_4^+	Nb	Cl	1.07	$NH_3NH_2^+$	Ta	Cl	1.23
NH_4^+	Mo	Cl	1.15	$NH_3NH_2^+$	W	Cl	1.18
NH_4^+	Rh	Cl	1.15	$NH_3NH_2^+$	Re	Cl	1.19
NH_4^+	Hf	Cl	1.17	$NH_3NH_2^+$	Bi	Cl	1.22
NH_4^+	Ta	Cl	1.18	$CH(NH_2)_2^+$	V	Cl	1.35
NH_4^+	W	Cl	1.16	$CH(NH_2)_2^+$	Ti	Cl	1.29
NH_4^+	Re	Cl	1.19	$C_2NH_6^+$	Pd	Cl	1.58
NH_3OH^+	Pd	Cl	1.32	$C_2NH_6^+$	Hg	Cl	1.56
NH_3OH^+	Hg	Cl	1.56	$C_2NH_6^+$	V	Cl	1.45
NH_3OH^+	V	Cl	1.24	$C_2NH_6^+$	Ti	Cl	1.34
NH_3OH^+	Ti	Cl	1.06	$C_2NH_6^+$	Ag	Cl	1.40
NH_3OH^+	Ag	Cl	1.18	$C_2NH_6^+$	Sc	Cl	1.32
NH_3OH^+	Sc	Cl	1.10	$C_2NH_6^+$	Y	Cl	1.16
NH_3OH^+	Y	Cl	1.07	$C_2NH_6^+$	Zr	Cl	1.17
NH_3OH^+	Zr	Cl	1.06	$C_2NH_6^+$	Nb	Cl	1.13
NH_3OH^+	Nb	Cl	1.07	$C_2NH_6^+$	Mo	Cl	1.39
NH_3OH^+	Mo	Cl	1.14	$C_2NH_6^+$	Rh	Cl	1.43
NH_3OH^+	Rh	Cl	1.14	$C_2NH_6^+$	Sb	Cl	1.25
NH_3OH^+	Sb	Cl	1.16	$C_2NH_6^+$	Hf	Cl	1.19
NH_3OH^+	Hf	Cl	1.13	$C_2NH_6^+$	Ta	Cl	1.22
NH_3OH^+	Ta	Cl	1.20	$C_2NH_6^+$	W	Cl	1.40
NH_3OH^+	W	Cl	1.14	$C_2NH_6^+$	Re	Cl	1.45
NH_3OH^+	Re	Cl	1.15	$C_2NH_6^+$	Bi	Cl	1.26
NH_3OH^+	Bi	Cl	1.17	$C_2OH_5^+$	Pd	Cl	1.51
$CH_3NH_3^+$	Pd	Cl	1.32	$C_2OH_5^+$	Hg	Cl	1.51
$CH_3NH_3^+$	Hg	Cl	1.55	$C_2OH_5^+$	V	Cl	1.36
$CH_3NH_3^+$	V	Cl	1.25	$C_2OH_5^+$	Ti	Cl	1.26
$CH_3NH_3^+$	Ti	Cl	1.07	$C_2OH_5^+$	Ag	Cl	1.31
$NH_3NH_2^+$	Pd	Cl	1.35	$C_2OH_5^+$	Sc	Cl	1.23

A	B	X	E_g^{GBR} (eV)	A	B	X	E_g^{GBR} (eV)
$C_2OH_5^+$	Y	Cl	1.08	$(CH_2)_3NH_2^+$	Hg	I	1.13
$C_2OH_5^+$	Zr	Cl	1.08	$(CH_2)_3NH_2^+$	Ag	I	1.12
$C_2OH_5^+$	Nb	Cl	1.06	$(CH_2)_3NH_2^+$	Sc	I	1.12
$C_2OH_5^+$	Mo	Cl	1.30	$(CH_2)_3NH_2^+$	Y	I	1.09
$C_2OH_5^+$	Rh	Cl	1.35	$(CH_2)_3NH_2^+$	Zr	I	1.10
$C_2OH_5^+$	Sb	Cl	1.17	$(CH_2)_3NH_2^+$	Nb	I	1.03
$C_2OH_5^+$	Hf	Cl	1.11	$(CH_2)_3NH_2^+$	Mo	I	1.37
$C_2OH_5^+$	Ta	Cl	1.15	$(CH_2)_3NH_2^+$	In	I	1.19
$C_2OH_5^+$	W	Cl	1.30	$(CH_2)_3NH_2^+$	Sb	I	1.08
$C_2OH_5^+$	Re	Cl	1.36	$(CH_2)_3NH_2^+$	Hf	I	1.20
$C_2OH_5^+$	Bi	Cl	1.17	$(CH_2)_3NH_2^+$	Ta	I	1.19
NH_4^+	Mn	Br	1.14	$(CH_2)_3NH_2^+$	W	I	1.40
NH_4^+	In	Br	1.18	$(CH_2)_3NH_2^+$	Tl	I	1.36
NH_3OH^+	Mn	Br	1.01	$(CH_2)_3NH_2^+$	Bi	I	1.20
NH_3OH^+	In	Br	0.92	$CH(NH_2)_2^+$	Tm	I	1.31
$CH_3NH_3^+$	Mn	Br	1.24	$CH(NH_2)_2^+$	Yb	I	1.38
$NH_3NH_2^+$	Mn	Br	1.27	$C_3N_2H_5^+$	Hg	I	1.43
$NH_3NH_2^+$	Cd	Br	1.02	$C_3N_2H_5^+$	Ag	I	1.55
$NH_3NH_2^+$	In	Br	1.06	$C_3N_2H_5^+$	Sc	I	1.54
$(CH_2)_3NH_2^+$	Y	Br	1.32	$C_3N_2H_5^+$	Y	I	1.37
$(CH_2)_3NH_2^+$	Zr	Br	1.33	$C_3N_2H_5^+$	Zr	I	1.37
$(CH_2)_3NH_2^+$	Sb	Br	1.53	$C_3N_2H_5^+$	Nb	I	1.36
$(CH_2)_3NH_2^+$	Hf	Br	1.48	$C_3N_2H_5^+$	Mo	I	1.58
$(CH_2)_3NH_2^+$	Bi	Br	1.58	$C_3N_2H_5^+$	In	I	1.42
$CH(NH_2)_2^+$	Mn	Br	0.98	$C_3N_2H_5^+$	Sb	I	1.38
$CH(NH_2)_2^+$	Tm	Br	1.51	$C_3N_2H_5^+$	Hf	I	1.39
$C_2NH_6^+$	Mn	Br	1.30	$C_3N_2H_5^+$	Ta	I	1.41
$C_2NH_6^+$	Sn	Br	1.22	$C_3N_2H_5^+$	W	I	1.59
$C_2NH_6^+$	In	Br	0.97	$C_3N_2H_5^+$	Tl	I	1.56
$C_2OH_5^+$	Mn	Br	1.17	$C_3N_2H_5^+$	Bi	I	1.42
$C_2OH_5^+$	In	Br	0.90	$(CH_3)_2NH_2^+$	Y	I	1.39
$C_2OH_5^+$	Sn	Br	1.10	$(CH_3)_2NH_2^+$	Zr	I	1.44
$C_2OH_5^+$	Pb	Br	1.53	$(CH_3)_2NH_2^+$	Nb	I	1.55
NH_4^+	Tm	I	1.41	$(CH_3)_2NH_2^+$	Sb	I	1.37
NH_4^+	Yb	I	1.52	$(CH_3)_2NH_2^+$	Hf	I	1.50
NH_3OH^+	Tm	I	1.39	$(CH_3)_2NH_2^+$	Bi	I	1.45
NH_3OH^+	Yb	I	1.48	$NC_4H_8^+$	Y	I	1.52
$CH_3NH_3^+$	Tm	I	1.45	$NC_4H_8^+$	Zr	I	1.55
$CH_3NH_3^+$	Yb	I	1.54	$NC_4H_8^+$	Sb	I	1.51
$NH_3NH_2^+$	Tm	I	1.45	$NC_4H_8^+$	Hf	I	1.57
$NH_3NH_2^+$	Yb	I	1.54	$NC_4H_8^+$	Bi	I	1.56
$(CH_2)_3NH_2^+$	Cd	I	1.36	$(NH_2)_3C^+$	Hg	I	1.51

A	B	X	E_g^{GBR} (eV)	A	B	X	E_g^{GBR} (eV)
$(NH_2)_3C^+$	Ag	I	1.52	$C_2NH_6^+$	Pb	I	1.52
$(NH_2)_3C^+$	Sc	I	1.47	$C_2OH_5^+$	Tm	I	1.41
$(NH_2)_3C^+$	Y	I	1.21	$C_2OH_5^+$	Yb	I	1.50
$(NH_2)_3C^+$	Zr	I	1.14	$C_2OH_5^+$	Sn	I	1.01
$(NH_2)_3C^+$	Nb	I	1.44	$C_2OH_5^+$	Pb	I	1.47
$(NH_2)_3C^+$	Mo	I	1.53	$C_3OH_7^+$	Y	I	1.59
$(NH_2)_3C^+$	In	I	1.58	$C_3OH_7^+$	Zr	I	1.59
$(NH_2)_3C^+$	Sb	I	1.18	$C_4NH_{10}^+$	Y	I	1.35
$(NH_2)_3C^+$	Hf	I	1.52	$C_4NH_{10}^+$	Zr	I	1.56
$(NH_2)_3C^+$	Ta	I	1.56	$C_4NH_{10}^+$	Sb	I	1.39
$(NH_2)_3C^+$	W	I	1.56	$C_4NH_{10}^+$	Tl	I	1.59
$(NH_2)_3C^+$	Tl	I	1.46	$C_4NH_{10}^+$	Bi	I	1.47
$(NH_2)_3C^+$	Bi	I	1.31	$C_4OH_9^+$	Zr	I	1.58
$(CH_3)_3NH^+$	Y	I	1.57	$C_4OH_9^+$	Sb	I	1.56
$(CH_3)_3NH^+$	Sb	I	1.36	$C_4OH_9^+$	Bi	I	1.59
$(CH_3)_3NH^+$	Tl	I	1.59	$C_5NH_{12}^+$	Y	I	1.56
$(CH_3)_3NH^+$	Bi	I	1.49	$C_5NH_{12}^+$	Zr	I	1.56
$(CH_3)_2CHNH_3^+$	Y	I	1.57	$C_5NH_{12}^+$	Nb	I	1.59
$(CH_3)_2CHNH_3^+$	Zr	I	1.59	$C_5NH_{12}^+$	Sb	I	1.56
$(CH_3)_2CHNH_3^+$	Nb	I	1.57	$C_5NH_{12}^+$	Tl	I	1.49
$(CH_3)_2CHNH_3^+$	Sb	I	1.55	$C_6NH_{14}^+$	Y	I	1.59
$(CH_3)_2CHNH_3^+$	Tl	I	1.55	$C_6NH_{14}^+$	Nb	I	1.59
$(CH_3)_2CHNH_3^+$	Bi	I	1.42	$C_6NH_{14}^+$	Tl	I	1.49
$C_2NH_6^+$	Tm	I	1.46	$C_6OH_{13}^+$	Y	I	1.59
$C_2NH_6^+$	Yb	I	1.55	$C_6OH_{13}^+$	Nb	I	1.59
$C_2NH_6^+$	Sn	I	1.08	$C_6OH_{13}^+$	Tl	I	1.49

T_f and O_f is tolerance factor and octahedral factor respectively. E_g^{GBR} is the bandgap predicted from GBR model.

Supplementary Notes

Supplementary Note 1. In Supplementary Table 1, the ionic polarizability, HOMO and LUMO for the A-sites molecules are obtained using Amsterdam Density Functional program package (ADF2013).[19,20] All calculations are carried out by using the PBE functional with the triple-zata plus polarization (TZP)[21] basis set. The protonated molecules' optimizations are done without any symmetry constraint before exploring electronic property.

Supplementary Note 2. For feature screening procedure, we used a method similar to the 'last-place elimination', as shown in Supplementary Fig. 3a. Firstly, 30 initial features are ranked by GBR algorithm according to the relative importance. Then, we remove the least important feature (*i.e.*, the 30th feature) out of the whole feature set. The remaining 29 features constitute a new feature set for the next step feature selection. Repeatedly, we rank the rest of features and remove the least important one. We record the model score (R^2) of trained ML model during each selection step and find that the ML model shows the best performance when the feature set includes 14 features, as is shown in Supplementary Fig. 3b. Moreover, it clearly shows when the number of features reaches 14, the addition of features has little impact on the prediction performance of the ML model. In other words, the rest 16 features removed have little effect on the bandgap of HOIPs.

Supplementary Note 3. As is shown in Supplementary Fig. 6, GBR algorithm has an advantage in terms of R^2 and MSE. When predicting unknown data, the performances of DTR and MLPR algorithm are not stable (the standard deviations of their R^2 are large). Although SVR, KRR and GPR have no standard deviations, their MSE is a little larger than GBR algorithm. As a result, we chose GBR algorithm, whose performance is the best among the six algorithms.

On the other hand, we compared GBR algorithm with other five algorithms according to R^2. We found that all of their P values (Sig.) are equal to zero (< 0.05) after test of normality for five paired samples' R^2 differences (Supplementary Table 2).

It demonstrates that their R^2 differences are all normally distributed and we are able to apply paired samples t test to their R^2 values.

As shown in Supplementary Table 3, the mean R^2 differences between GBR algorithm and other five ML algorithms are all positive, which means the prediction performance of GBR algorithm is better in general. What's more, the P values (Sig. (2-tailed)) of five pairs are all equal to zero (< 0.05), which shows that GBR algorithm is significantly different from the other five algorithms. This result can also be obtained from the 95% confidence interval (CI) of the average difference. If zero is not included in 95% CI, $P<0.05$. In five paired samples t tests, none of them contains zero.

Supplementary Note 4. As shown in Supplementary Fig. 8, 1669 HOIPs are screened out from the total 5158 HOIPs with ML predicted bandgap according to structural stability ($0.5 < T_f < 1.2$, $0.4 < O_f < 0.7$) firstly. Then, the selected HOIPs are divided into four parts by bandgap. For solar cells, HOIPs with bandgap between 0.9 eV and 1.6 eV are ideal candidates. Therefore, 218 HOIPs with proper bandgap are selected. Subsequently, these candidates are distinguished using X-site elements as screen standard. Here, we only focus on Br-based HOIPs (22 $ABBr_3$). Additionally, magnetism normally has significant influences on electronic structures of materials and toxicity of HOIPs will block widespread commercial application, therefore, we further exclude the magnetic and/or toxic compounds in 22 HOIPs. Finally, 6 orthorhombic HOIPs are screened out for further thermal and environmental stability evaluation and electronic property are further investigated by using DFT.

Supplementary Note 5. To clear evaluate the effect of SOC on their band gap, we calculated the band structures of the six selected HOIPs at the PBE+SOC level of theory. As is shown in Supplementary Fig. 11, the SOC effect is not pronounced and the band structures obtained by PBE and PBE+SOC are very similar, therefore we neglected SOC effect on these six HOIPs.

Supplementary Methods

Model evaluation. Evaluating the training model performance is the key to the accurate prediction. The training model is based on a subset of the whole data, known as training data, and the training model will be used to predict other new data after training. Different ML tasks have different performance evaluation indexes. Here, we choose three indexes including coefficient of determination, mean squared error and Pearson coefficient to estimate the prediction error[22].

The coefficient of determination (R^2), employed to evaluate the model accuracy (goodness of fit), is defined as

$$R^2 = 1 - \frac{\sum_i \left(y_i^{true} - y_i^{pred} \right)^2}{\sum_i \left(y_i^{true} - \overline{y}_i^{true} \right)^2} \tag{1}$$

where y is the bandgap value. The closer to 1 the value of R^2, the better fitting degree of prediction values the regression line.

The mean squared error (MSE) represents the mean difference between the predicted values and the real values, defined as

$$MSE = \frac{1}{N} \sum_i^N \left| y_i^{true} - y_i^{pred} \right| \tag{2}$$

The correlation between predictive value and real value can be reflected by Pearson coefficient (r), as

$$r^2 = \frac{\sum_i \left(y_i^{true} - \overline{y}_i^{true} \right)\left(y_i^{pred} - \overline{y}_i^{pred} \right)}{\sqrt{\sum_i \left(y_i^{true} - \overline{y}_i^{true} \right)^2 \sum_i \left(y_i^{pred} - \overline{y}_i^{pred} \right)^2}} \tag{3}$$

The value of r is between -1 and +1. If r is larger than zero, it indicates that the two variables are positively correlated, that is, the larger of one variable is, the larger of the other variable will be. If r is less than zero, it suggests that the two variables are negatively correlated. In addition, the greater the absolute value of r, the stronger the correlation.

Density functional theory. All first-principles calculations for selected HOIPs were carried out using the projector-augmented wave (PAW) method with the generalized gradient approximation (GGA), implemented in the Vienna Ab initio Simulation Package package[23]. The exchange-correlation functional was described by Perdew–Burke–Ernzerh (PBE)[24] functional considering the PBE method is more consistent with the experimental results for the HOIP materials due to fortuitous error–error offset[25, 26]. The cutoff energy for the plane-wave basis was set as 520 eV. Furthermore, the DFT-D3 method was adopted for the van der Waals correction[27]. The structure optimization process ended until an energy convergence threshold of 10^{-5} eV and atomic force less than 0.01 eV/Å. The initial HOIP structures in a ($\sqrt{2} \times \sqrt{2} \times 2$) unit cell were constructed within periodic boundary condition. The Brillouin zone integration was performed using a 4 × 3 × 4 *k*-point mesh for the orthorhombic phase.

Ab initio molecular dynamics (AIMD) simulations were performed to confirm dynamics stability of the selected materials, which is in supercells of $2\sqrt{2} \times 2\sqrt{2} \times 2$ of unit cell. The entire MD simulation lasted 5 ps with the step of 1 fs. The temperature was controlled at 300K by using the Nosé-Hoover method[28, 29].

The adsorptions of H_2O/O_2 on the (001) surface of the HOIP structures were investigated and the H_2O/O_2 binding energy E_{ads} was defined as: $E_{ads} = E_{HOIP\text{-}H2O/O2} - E_{HOIP} - E_{H2O/O2}$, where $E_{HOIP\text{-}H2O/O2}$, E_{HOIP} and $E_{H2O/O2}$ are the total energies of the H_2O/O_2-adsorbed HOIP structures, the HOIP structures and H_2O/O_2 respectively[30]. It was calculated in supercells with a vacuum space larger than 18 Å above the structure along the z-axis. Initially, one water molecule (oxygen molecule) was put at the top of the organic molecule on ABr-terminated surface.

Supplementary references

1. Smola, A. J. & Schölkopf, B. A Tutorial on Support Vector Regression. *Stat.Comput.* **14**, 199-222 (2004).
2. Murphy, K. P., *Machine Learning: A Probabilistic Perspective CH. 14.4.3* (MIT Press, Massachusetts, 2012).
3. Rasmussen, C. E. & Williams, C. K. I., *Gaussian Processes for Machine Learning* (MIT Press, Massachusetts, 2006).
4. Hastie, T., Tibshirani, R. & Friedman, J., *Elements of Statistical Learning* (Springer. Press, New York, 2009).
5. Rumelhart, D. E., Hinton, G. E. & Williams, R. J., Learning representations by back-propagating errors. *Nature* **323**, 533-536 (1986).
6. Montavon, G., Orr, G. B. & Müller, K. R., *Neural Networks: Tricks of the Trade*. (Springer. Press, New York, 1998).
7. Shannon, R. D. Revised effective ionic radii and systematic studies of interatomic distances in halides and chalcogenides. *Acta. Crystallogr. A* **32**, 751–767 (1976).
8. Jia, Y. Q. Crystal radii and effective ionic radii of the rare earth ions. *J. Solid State Chem.* **95**, 184-187 (1991).
9. Kieslich, G., Sun, S. & Cheetham, A. K. An extended Tolerance Factor approach for organic-inorganic perovskites. *Chem. Sci.* **6**, 3430-3433 (2015).
10. Goldschmidt, V. M. Die gesetze der krystallochemie. *Naturwissenschaften* **14**, 477-485 (1926).
11. Kieslich, G., Sun, S. & Cheetham, A. K. Solid-state principles applied to organic–inorganic perovskites: new tricks for an old dog. *Chem. Sci.* 5, 4712-4715 (2014).
12. Becker, M., Klüner, T. & Wark, M. Formation of hybrid ABX_3 perovskite compounds for solar cell application: first-principles calculations of effective ionic radii and determination of tolerance factors. *Dalton Trans.* **46**, 3500-3509 (2017).
13. Wang, J. W., Guo, Q. T. & Kleppa, O. J. Standard enthalpies of formation of some T alloys with Group VIII elements (Co, Ni, Ru, Rh, Pd, Ir and Pt), determined by high-temperature direct synthesis calorimetry. *J. Alloy. Compd.* **313**, 77–84 (2000).

14. Martynov, A. I. & Batsanov, S. S. New approach to calculating atomic electronegativities. *Russ. J. Inorg. Chem.* **25**, 1737-1740 (1980).

15. Rabe, K. M. *et al.* Global multinary structural chemistry of stable quasicrystals, high-Tc ferroelectrics, and high-Tc superconductors. *Phys. Rev. B* **45**, 7650-7676 (1992).

16. Shevelko, V. P. & Ulantsev, A. D. Static multipole polarizability of atoms and ions in the Thomas-Fermi model. *J. Russ. Laser. Res.* **15**, 529-545 (1994).

17. Martin, W. C. & Wiese, W. L., in *Atomic, Molecular, and Optical Phsics Handbook, Drake, G. W. F., Ed.,* (AIP Press, New York, 1996).

18. Haynes, W. M., Lide, D. R. & Bruno, T. J. *CRC Handbook of Chemistry and Physics*. (CRC Press, New York, 2015).

19. Guerra, C. F., Snijders, J. G., te Velde, G. & Baerends, E. J., Towards an Order-N DFT Method. *Theor. Chem. Acc.* **99**, 391-403 (1998).

20. te Velde, G. *et.al.* Chemistry with ADF. *J. Comput. Chem.* **22**, 931-967 (2001).

21. Van Lenthe, E. & Baerends, E. J., Optimized Slater-Type Basis Sets for the Elements 1-118. *J. Comput. Chem.* **24**, 1142-56 (2003).

22. Isayev, O., Oses, C., Toher, C., Gossett, E., Curtarolo, S. & Tropsha, A. Universal fragment descriptors for predicting properties of inorganic crystals. *Nat. Commun.* **8**, 15679 (2017).

23. Kresse, G. & Furthmüller, J. Efficiency of Ab-Initio Total Energy Calculations for Metals and Semiconductors Using a Plane-Wave Basis Set. *Comput. Mater. Sci.* **6,** 15-50 (1996).

24. Perdew, J. P., Burke, K. & Ernzerhof, M. Generalized gradient approximation made simple. *Phys. Rev. Lett.* **77**, 3865 (1996).

25. Motta, C., El-Mellouhi, F., Kais, S., Tabet, N., Alharbi, F. & Sanvito, S. Revealing the role of organic cations in hybrid halide perovskite $CH_3NH_3PbI_3$, *Nat. Commun.* **6,** 7026 (2015).

26. Colella, S. *et al.* $MAPbI_{3-x}Cl_x$ mixed halide perovskite for hybrid solar cells: the role of chloride as dopant on the transport and structural properties, *Chem. Mater.* **25**, 4613-4618 (2013).

27. Lee, K., Murray, É. D., Kong, L., Lundqvist, B. I. & Langreth, D. C. Higher-accuracy van der Waals density functional. *Phys. Rev. B* **82**, 081101(R) (2010).

28. Nose, S. A unified formulation of the constant temperature molecular dynamics methods. *J. Chem. Phys.* **81**, 511−519 (1984).

29. Hoover, W. G. Canonical dynamics: Equilibrium phase-space distributions. *Phys. Rev. A* **31**, 1695-1697 (1985).

30. Zhang, L. & Sit, P. H L. Ab initio study of the role of oxygen and excess electrons in the degradation of $CH_3NH_3PbI_3$. *J. Mater. Chem. A* **5**, 9042-9049 (2017).
